# Blood Leukocyte Ratios as Predictive Markers of Chronic Enteropathy Phenotypes in Cats

**DOI:** 10.3390/vetsci12070613

**Published:** 2025-06-24

**Authors:** Alexandros O. Konstantinidis, Katerina K. Adamama-Moraitou, Ashley Griggs, Margaret L. Musser, Ariel S. Nenninger, Nektarios Soubasis, Dimitra Pardali, Mathios E. Mylonakis, Albert E. Jergens

**Affiliations:** 1 Companion Animal Clinic, School of Veterinary Medicine, Faculty of Health Sciences, Aristotle University of Thessaloniki, 54627 Thessaloniki, Greece; kadamama@vet.auth.gr (K.K.A.-M.); nsouba@vet.auth.gr (N.S.); mmylonak@vet.auth.gr (M.E.M.); 2 College of Liberal Arts and Science, Iowa State University, Ames, IA 50014, USA; agriggs1@iastate.edu; 3 Department of Veterinary Clinical Sciences, College of Veterinary Medicine, Iowa State University, Ames, IA 50011-1250, USA; mmusser@iastate.edu (M.L.M.); ajergens@iastate.edu (A.E.J.); 4 Department of Veterinary Pathology, College of Veterinary Medicine, Iowa State University, Ames, IA 50011, USA; arieln@iastate.edu; 5 Diagnostic Laboratory, School of Veterinary Medicine, Faculty of Health Sciences, Aristotle University of Thessaloniki, 54627 Thessaloniki, Greece; dpardali@vet.auth.gr

**Keywords:** alimentary lymphoma, biomarker, chronic enteropathy, feline, inflammatory bowel disease, leukocytes

## Abstract

This retrospective study assessed the potential of blood leukocyte ratios as diagnostic biomarkers in cats with chronic enteropathies (CE). Absolute neutrophil-to-lymphocyte (NLR), neutrophil-to-monocyte (NMR), and lymphocyte-to-monocyte (LMR) ratios were calculated from the hematological profiles of 221 cats, including healthy controls (*n* = 73), and those diagnosed with food-responsive enteropathy (FRE, *n* = 59), steroid-responsive enteropathy (SRE, *n* = 56), or small cell lymphoma (SCL, *n* = 33). SCL cats had higher NLRs than SRE (*p* = 0.002) and FRE (*p* = 0.028), and lower LMRs than SRE (*p* = 0.012) and FRE (*p* = 0.001). Healthy cats had lower NLRs compared to the FRE (*p* < 0.001), SRE (*p* < 0.001), and SCL (*p* < 0.001) cats and higher LMRs compared to the FRE (*p* < 0.001), SRE (*p* < 0.001), and SCL (*p* < 0.001) cats. Receiver operating characteristic (ROC) curve analysis demonstrated that NLR ≥ 11.6 differentiated SCL from SRE with 87.5% specificity but low sensitivity (39.4%). NMR ≥ 34.5 distinguished FRE from SRE with 52.5% sensitivity and 69.6% specificity. LMR ≥ 3.72 differentiated SRE from SCL with 67.9% sensitivity and 60.6% specificity. Although significant differences in leukocyte ratios were observed among groups, their diagnostic accuracy in differentiating CE phenotypes was suboptimal. These findings suggest that the utility of NLR, NMR, and LMR as standalone diagnostic tools is limited.

## Simple Summary

This retrospective study assessed whether blood leukocyte ratios could help differentiate between types of feline chronic enteropathies (CE). Absolute neutrophil-to-lymphocyte (NLR), neutrophil-to-monocyte (NMR), and lymphocyte-to-monocyte (LMR) ratios were calculated from the hematological data of 221 cats: 73 healthy controls and 148 cats diagnosed with food-responsive enteropathy (FRE), steroid-responsive enteropathy (SRE), or small cell lymphoma (SCL). Cats with SCL had significantly higher NLRs and lower LMRs compared to those with FRE and SRE. Healthy cats showed lower NLRs and higher LMRs than all CE subgroups. While these ratios differed between groups, their ability to accurately classify disease was suboptimal. NLR ≥ 11.6 distinguished SCL from SRE with 87.5% specificity but only 39.4% sensitivity. NMR ≥ 34.5 differentiated FRE from SRE with 52.5% sensitivity and 69.6% specificity, and LMR ≥ 3.72 separated SRE from SCL with 67.9% sensitivity and 60.6% specificity. Overall, blood leukocyte ratios reflect underlying inflammation but lack sufficient accuracy to serve as standalone diagnostic markers for feline CE phenotypes.

## 1. Introduction

Feline chronic enteropathies (CE) are a group of gastrointestinal (GI) disorders that cause intermittent or persistent GI signs and variable degrees of intestinal inflammation [1,2,3,4,5,6]. Feline CEs are classified retrospectively based on sequential response to treatment as food-responsive enteropathy (FRE), steroid-responsive enteropathy (SRE), often referred to as idiopathic inflammatory bowel disease (IBD), and intestinal small cell lymphoma (SCL) [5,6]. Food-responsive enteropathy (FRE) cases are characterized by rapid clinical remission following a trial with a therapeutic diet, such as novel protein, hydrolyzed protein, or highly digestible diet [7,8,9]. Although the true prevalence remains uncertain, FRE is estimated to account for approximately 50% of cases. SRE cases are characterized by a lack of response to dietary intervention but achieving complete or partial clinical remission with immunosuppressive doses of glucocorticoids [6,10,11,12]. SCL is the most common form of intestinal lymphoma in cats, accounting for up to 75% of all feline GI lymphomas. It is characterized by small, mature lymphocytes with low mitotic activity, and its clinical progression is typically slow [3,11,13,14]. SRE and SCL are most common in middle-aged and older cats and should be differentiated from other causes of primary and secondary enteropathies through diagnostic testing [5,6,15]. Several retrospective studies have shown that cats with FRE, SRE, and SCL share signalment, history, and clinical features [6]. Furthermore, progression of SRE to SCL over months to years is often suspected in cats with SCL and a previous history of SRE or FRE based on the coexistence of inflammatory and neoplastic lesions [3,16]. While the diagnosis of FRE is based on dietary trials, intestinal biopsies with proper histopathologic evaluation are necessary to differentiate between lesions of SCL and SRE. When histopathology cannot differentiate SCL from SRE, immunohistochemistry (IHC) confirming a predominant mucosal T-cell population and PCR assay for antigen receptor rearrangement (PARR) have been successfully used for diagnosis [17]. Thus, the current recommendations for distinguishing SCL from SRE include a combination of histopathology, IHC, and PARR [10]. However, even this diagnostic algorithm has recently been questioned as to the accuracy of the final diagnosis [18].

Easily attainable, efficacious, and cost-effective diagnostic markers to differentiate between FRE, SRE, and SCL would be of value to clinicians [19]. Numerous biomarkers have been proposed to differentiate between SRE and SCL in cats [6,10,20,21,22]. However, none of these have replaced the need for histopathologic and molecular evaluation of intestinal biopsies to date.

A complete blood count (CBC) is routinely performed as part of a standard diagnostic evaluation in sick cats. Characteristic leukogram changes expressed as leukogram ratios (e.g., neutrophil-to-lymphocyte ratio [NLR]) are useful hematological markers of inflammation and stress in multiple species. In human medicine, these ratios have shown diagnostic and prognostic value in various inflammatory and neoplastic conditions [23,24,25,26,27]. Specifically, in GI diseases, leukocyte ratios such as the NLR and the lymphocyte-to-monocyte ratio (LMR) have been employed as diagnostic and prognostic markers in both inflammatory bowel disease (IBD) and GI neoplasia. Specifically, NLR in IBD has demonstrated utility for disease differentiation, particularly in distinguishing Crohn’s disease from ulcerative colitis, monitoring of clinical activity, prediction of treatment response, and assessment of complication risk, while LMR has been shown to correlate strongly with disease activity and endoscopic severity, particularly in ulcerative colitis [28,29,30,31,32,33,34,35,36]. In addition, NLR, along with other inflammatory markers such as platelet-to-lymphocyte ratio (PLR), has demonstrated significant diagnostic and prognostic value in colorectal cancer, contributing to early disease recognition and accurate staging [37]. Elevated NLR is associated with more advanced disease and poorer survival, making it a useful tool for identifying high-risk patients who may benefit from adjuvant therapies [38]. In gastric cancer, the diagnostic and screening potential of NLR and PLR is also noteworthy, as their strong discriminatory capacity supports their use as non-invasive biomarkers for early detection [39]. NLR has also been evaluated and proven useful for diagnosing different diseases in dogs and cats [40,41,42,43,44]. Recent studies have explored the clinical significance of NLR and other leukocyte ratios as potential biomarkers in dogs with chronic enteropathy (CE), suggesting they may aid in the subclassification of CE phenotypes, provide insights into disease severity, and help predict treatment response. However, findings have been inconsistent, and further well-designed studies are needed to clarify their diagnostic and prognostic utility [44,45,46,47]. Similar studies have not been performed in cats with CE.

The aim of this study was to evaluate and compare NLR, neutrophil-to-monocyte ratio (NMR), and lymphocyte-to-monocyte ratio (LMR) as diagnostic biomarkers in healthy cats and cats with CE. We hypothesized that these ratios would (1) exhibit significant differences among cats with FRE, SRE, and SCL as compared to healthy control (HC) cats; (2) demonstrate correlations with different clinicopathologic parameters in cats with CE; and (3) enable differentiation between the different phenotypes of CE (e.g., FRE, SRE, and SCL).

## 2. Materials and Methods

### 2.1. Healthy Control Cats

For the HC group, the medical archives of client-owned cats brought to the College of Veterinary Medicine, Iowa State University (ISU) and the School of Veterinary Medicine, Aristotle University of Thessaloniki (AUTh) for routine wellness care visits between 2010 and 2022 were retrospectively reviewed. Cats that underwent thorough assessments, including history taking, clinical examination, CBC, serum biochemistry profile, urinalysis, and serologic screening for feline immunodeficiency virus (FIV) and feline leukemia virus (FeLV) status, confirming their healthy status, were eligible for entering the study. Cats in the HC group were required not to have shown GI or other signs or been exposed to medications (apart from routine prophylactic antiparasitic treatments) for at least 6 months preceding their presentation.

### 2.2. Cats with Chronic Enteropathies

The medical records of cats from the ISU and the AUTh between 2010 and 2022 were retrospectively reviewed to identify cats diagnosed with CE. The study population consisted of three groups of cats that were diagnosed at the ISU or AUTh Small Animal Teaching Hospitals with FRE, SRE, or SCL.

Clinical disease activity was assessed at presentation for each cat using a modified version of the feline chronic enteropathy activity index (FCEAI) [2]. This modified version evaluates the clinical parameters of gastroenteritis (attitude/activity, appetite, vomiting, diarrhea, and weight loss) to define disease activity. Each of the five clinical parameters were graded from 0 to 3 (0 = normal, 1 = slightly abnormal, 2 = moderately abnormal, and 3 = severely abnormal) and summed, yielding a cumulative activity score. All FCEAI scores were derived from the medical records or calculated.

The initial diagnostic investigations for all cats with suspected CE included a thorough clinical examination, a CBC, serum biochemistry profile, urinalysis, fecal parasitology screen, feline pancreatic lipase immunoreactivity (fPLI), abdominal radiographs +/− abdominal ultrasound examination, and serologic screening for FIV and FeLV status. Serum folate and cobalamin were evaluated in some of the cats with suspected CE. Feline trypsin-like immunoreactivity (TLI), serum thyroxine (total T4), and pre- and post-prandial bile acids concentrations were assessed in some cats based on their history, physical examination findings, baseline laboratory test results, and diagnostic imaging findings. Cats with CE and clinical or laboratory evidence of clinically relevant comorbidities (e.g., kidney or liver disease, hyperthyroidism, and other neoplastic diseases) were excluded from the study. Cats with CE were excluded if they had clinical or laboratory evidence of concurrent disease (e.g., kidney or liver disease, hyperthyroidism, or other neoplastic diseases), if they tested positive for FIV or FeLV, or if they were receiving medical treatment for unrelated conditions, in order to minimize potential confounding effects on leukocyte parameters. Additional exclusion criteria, applicable to all cats included in the FRE, SRE, and SCL groups, included administration of antibiotics or immunosuppressive drugs within three weeks prior to presentation, incomplete medical records, and insufficient diagnostic information to confirm a diagnosis of CE or rule out other causes of chronic GI signs.

The FRE group consisted of cats that responded to one or more dietary trials with either limited ingredient, selected protein, or hydrolyzed protein diets [7,8,9], and that did not relapse during the following 6 months. The SRE and SCL groups included cats that failed to respond to a properly performed dietary trial. All cats in these two groups underwent either GI endoscopy to obtain GI mucosal biopsy specimens or laparotomy to acquire full thickness biopsy specimens from the stomach, duodenum, jejunum, ileum, and colon. Following overnight fixation in 10% formalin and routine histopathological processing, hematoxylin and eosin (H&E) stained tissues were evaluated for inflammatory, neoplastic, or other lesions by board-certified service pathologists. A diagnosis of mucosal inflammation (e.g., lymphoplasmacytic enteritis [LPE]) was made using previously published histopathologic criteria [48]. Cases with a histopathologic diagnosis of SCL or those with suspicion of underlying SCL underwent further diagnostic testing, including IHC (e.g., immunophenotyping using CD3+ antibodies to detect a predominant [>90%] population of T-lymphocytes) and/or PCR for PARR testing. Final diagnosis of SRE or SCL was established through the integration of results from H&E histopathology, IHC, and PARR.

### 2.3. Laboratory Measurements

EDTA-anticoagulated whole blood was used for CBC, which was performed at the ISU Clinical Pathology Laboratory (ADVIA 2120i Hematology System, Siemens Medical Solutions USA Inc., Malvern, PA, USA) for cases enrolled retrospectively at the ISU, and at the Diagnostic Laboratory of School of Veterinary Medicine, AUTh (ADVIA 120 Hematology System, Siemens Medical Solutions USA Inc., Malvern, PA, USA) for cases enrolled at the AUTh. Neutrophil, lymphocyte, and monocyte absolute counts were extracted from the routine hematologic profile. NLR was calculated by dividing the total neutrophil count by the total lymphocyte count, NMR was calculated by dividing the total neutrophil count by the total monocyte count, and LMR was calculated by dividing the total lymphocyte count by the total monocyte count, as previously described [44,46,47,49].

Biochemical parameters, including total protein (TP) and albumin (Alb), were measured using automated chemistry analyzers (Ortho VITROS 4600 chemistry system, Ortho TSB, Ortho Clinical Diagnostics, Raritan, NJ, USA for ISU cases; Clinical Chemistry Analyzer, FlexorE, Vital Scientific N.V., Dieren, The Netherlands for AUTh cases).

### 2.4. Statistical Analyses

Data were summarized by computing absolute and relative frequencies (%), indices of central tendency (median values), and indices of variability (minimum and maximum values). The association between quantitative parameters was examined by evaluating the statistical significance and the magnitude of Spearman’s *rho* (*ρ*) rank correlation coefficient. Spearman’s *ρ* was interpreted as indicating a very strong (0.8–1.0), strong (0.6–0.8), moderate (0.4–0.6), weak (0.2–0.4), or very weak (0–0.2) correlation [50]. The four groups of cats (FRE, SRE, SCL, HC) were compared relative to the distribution of quantitative parameters (cats’ age; modified FCEAI scores; white blood cell, neutrophil, lymphocyte, and monocyte counts; NLR, NMR and LMR; and serum Alb and TP) with the Kruskal–Wallis (K-W) test [51]. After a significant K-W result, pairs of groups were compared with the Mann–Whitney (M-W) test. A Chi-squared test (*χ*^2^-test) was performed for comparing the distribution of sexes (male, female) among the four groups of cats. A series of receiver operating characteristic (ROC) curve analyses calculated the sensitivity and specificity at optimum cut-off concentrations [as determined by the Youden index [49,52,53]] to differentiate (a) cats with FRE from those with SRE, (b) cats with FRE from those with SCL, and (c) cats with SRE from those with SCL. The area under the curve (AUC) was calculated to evaluate the diagnostic performance of the tested parameters. The AUC was categorized as low (0.5 < AUC ≤ 0.7), moderate (0.7 < AUC ≤ 0.9), or high (0.9 < AUC ≤ 1.0) [54]. In all non-parametric hypotheses testing procedures (K-W, M-W, and *χ*^2^), the observed significance level (*p*-value) was computed either with the Monte-Carlo simulation method (based on 10,000 resampling circles) or with the Exact method [55]. This method leads to safe and valid inferential conclusions, even in cases where the methodological presupposition and assumptions of the non-parametric tests are not fulfilled (e.g., random samples, independent measurements, symmetrical distributions, and absence of extreme outliers). Statistical analyses were accomplished with IBM SPSS Statistics v.29.0 (IBM Corp., Armonk, NY, USA) software enhanced with the module Exact Tests (for implementation of the Monte-Carlo and Exact methods). In all hypothesis testing procedures, the significance level was preset at *a* = 0.05 (*p* ≤ 0.05).

## 3. Results

### 3.1. Animals

It should provide a concise and precise description of the experimental results, their interpretation, as well as the experimental conclusions that can be drawn.

The HC group consisted of 73 healthy cats presented during the same time period as the CE cats (Table 1). The median age was 7 years (range: 1–15). The population consisted of 25 male (24 neutered) and 48 female cats (45 spayed). Seven different breeds of cats were included, with the majority being DSH (61/73, 83.6%).

**Figure 1:**
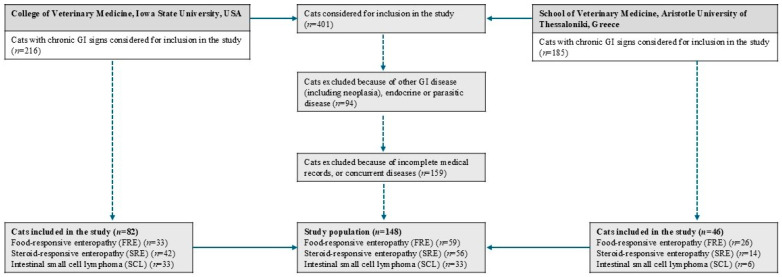
Inclusion flowchart for the evaluation of blood neutrophil-to-lymphocyte ratio (NLR), lymphocyte-to-monocyte ratio (LMR), and neutrophil-to-monocyte ratio (NMR) as a diagnostic markers in cats with chronic enteropathies. Of the 198 cats initially considered for inclusion in the study, 148 cats ultimately met the criteria for inclusion.

One hundred and forty-eight cats with CE were enrolled in the study; 59 cats with FRE, 56 cats with SRE, and 33 cats with SCL (Table 1 and Table S1) (Figure 1). The median age of the cats with SCL was 11 years (range: 6–16) and was significantly higher compared to the cats with FRE (median age: 8 years; range: 1–16) (*p* < 0.001) and SRE (median age: 8.5 years; range: 1.5–17) (*p* = 0.003) and the HC cats (*p* < 0.001). In addition, healthy cats were significantly younger compared to cats with SRE (*p* = 0.008) and cats with SCL (*p* < 0.001).

There was no significant difference in sex among the CE groups and healthy cats (*p* = 0.05). The whole population included 105 males and 116 females, with 102 (97.1%) sterilized males and 110 (94.8%) spayed females. No statistically significant differences were detected among the four groups relative to the distribution of sexes (*p* = 0.091). Cats with CE included twenty different breeds with DSH representing the most frequently represented breed in all three CE groups (Table 1).

Among the cats with SRE, diagnosis was reached based on histopathology alone in 49/56 cats, and on histopathology in conjunction with IHC in 4/56 cats or PARR in 3/56 cats. Among the cats with SCL included in the study, diagnosis was established through histopathology, with complementary IHC in 25/33 cats and PARR in 3/33 cases. Diagnosis was based solely on histopathology in only five cats (Table S1).

Among the cats included in the study, the modified FCEAI score was calculated at the time of diagnosis for 25 out of 59 (42.4%) of the FRE cases, 13 out of 56 (23.2%) of the SRE cases, and 6 out of 33 (18.2%) of the SCL cases, while for the remaining cases, it was calculated retrospectively. The modified FCEAI score of FRE cats (median: 2, range: 1–8) was significantly lower compared to the SRE (median: 4, range: 2–10) (*p* < 0.001) and the SCL (median: 5, range: 2–10) (*p* < 0.001) groups (Table 1). In addition, the SCL group had a significantly higher modified FCEAI score compared to the SRE group (*p* = 0.002).

### 3.2. Complete Blood Count and Leukocyte Ratios

**Figure 2:**
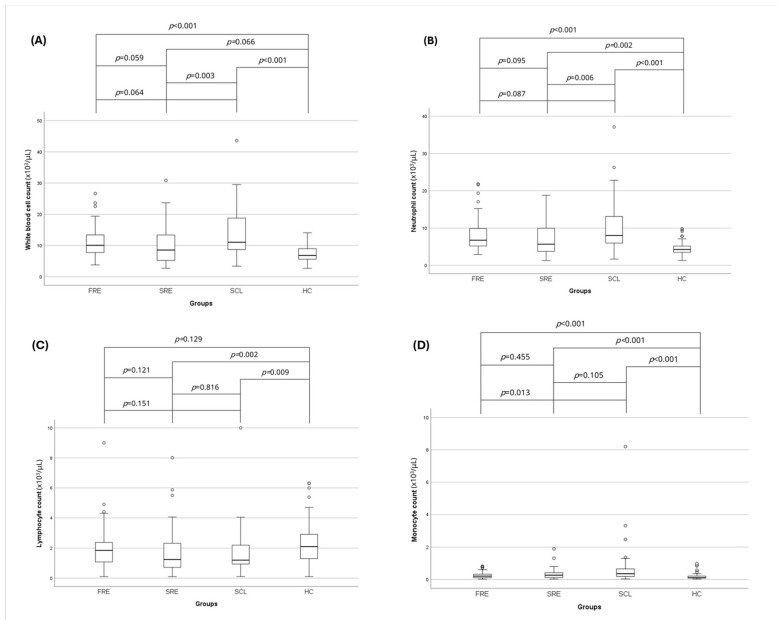
Box-plots comparing (**A**) white blood cell, (**B**) neutrophil, (**C**) lymphocyte, and (**D**) monocyte counts in HC cats (*n* = 73) and cats with FRE (*n* = 59), SRE (*n* = 56), and intestinal SCL (*n* = 33). Circles represent outliers. Statistical significance was defined as *p* < 0.05. FRE: food-responsive enteropathy; SRE: steroid-responsive enteropathy; SCL: small cell lymphoma; HC: healthy control cats.

The total white blood cell counts (WBC, ×10^3^/μL) of the HC group (median: 6.8; range: 2.67–14.07) were significantly lower compared to the FRE (median: 10.03; range: 3.79–26.63) (*p* < 0.001) and SCL (median: 11.02; range: 3.37–43.60) (*p* < 0.001) groups (Table 2) (Figure 2). In addition, the WBC of the SCL group was significantly higher compared to the SRE group (median: 8.54; range: 2.70–30.81) (*p* = 0.003). The neutrophil count (×10^3^/μL) of the HC group (median: 4.3; range: 1.3–9.9) was significantly lower compared to the FRE (median: 6.75; range: 2.90–21.84) (*p* < 0.001), SRE (median: 5.68; range: 1.30–18.80) (*p* = 0.002), and SCL (median: 8.01; range: 1.69–37.10), (*p* < 0.001) groups (Table 2) (Figure 2). In addition, the SCL group had a significantly higher neutrophil count compared to the SRE group (*p* = 0.006) (Table 2) (Figure 2). The HC group (median: 2.10; range 0.10–6.30) had a significantly higher lymphocyte count (×10^3^/μL) compared to the SRE (median: 1.25; range: 0.10–8.01) (*p* = 0.002) and SCL (median: 1.2; range: 0.10–10.00) (*p* = 0.009) groups (Table 2) (Figure 2). The monocyte count (×10^3^/μL) of the HC group (median: 0.10; range: 0.02–0.98) was significantly lower compared to the FRE (median: 0.20; range: 0.02–0.81) (*p* < 0.001), SRE (median: 0.26; range: 0.02–1.89) (*p* < 0.001), and SCL (median: 0.35; range: 0.03–8.20) (*p* < 0.001) groups (Table 2) (Figure 2). Finally, the monocyte count of the FRE group was significantly lower compared to the SCL group (*p* = 0.013) (Table 2) (Figure 2).

**Figure 3:**
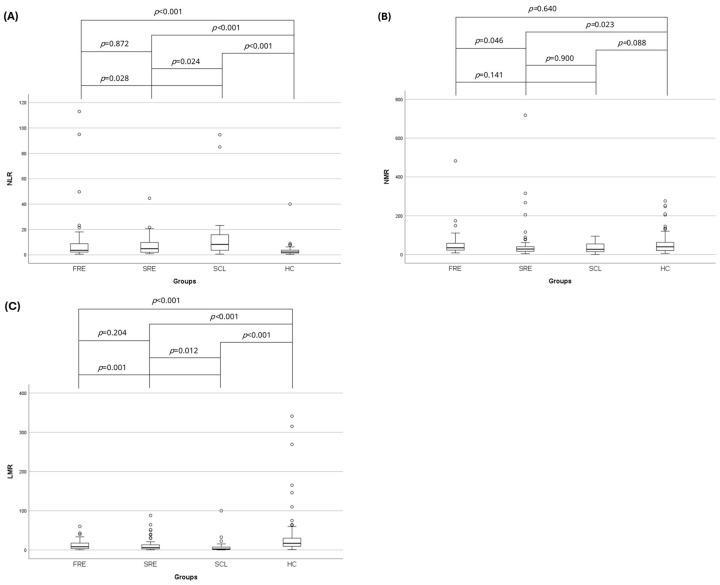
Box-plots comparing (**A**) NLR, (**B**) LMR, and (**C**) NMR in HC cats (*n* = 73) and cats with FRE (*n* = 59), SRE (*n* = 56), and intestinal SCL (*n* = 33). Circles represent outliers. Statistical significance was defined as *p* ≤ 0.05. NLR: neutrophil-to-lymphocyte ratio; LMR: lymphocyte-to-monocyte ratio; NMR: neutrophil-to-monocyte ratio; FRE: food-responsive enteropathy; SRE: steroid-responsive enteropathy; SCL: small cell lymphoma; HC: healthy control cats.

The NLR was significantly higher in the FRE (median: 3.64; range: 0.53–113.00) (*p* < 0.001), SRE (median: 4.84; range: 0.85–44.60) (*p* < 0.001) and SCL (median: 8.26; range: 0.57–94.71) (*p* = 0.001) groups compared to the HC group (median: 2.24; range: 0.38–40.00) (Table 2) (Figure 3). In addition, the NLR of the SCL group was higher compared to the FRE (*p* = 0.028) and SRE (*p* = 0.024) groups (Table 2) (Figure 3). The NMR was significantly lower in the SRE group (median: 28.34; range: 4.33–718.00) compared to the FRE (median: 35.00; range: 8.32–482.50) (*p* = 0.046) and HC groups (median: 39.76; range: 5.67–276.00) (*p* = 0.023) (Table 2) (Figure 3). LMR was significantly lower in the FRE (median: 8.32; range: 0.50–60.13) (*p* = 0.001), SRE (median: 6.00; range: 0.67–88.00) (*p* < 0.001), and SCL (median: 2.47; range: 0.28–100.00) (*p* < 0.001) groups compared to the HC group (median: 17.00; range: 1.00–341.00) (Table 2) (Figure 3). In addition, the LMR in the SCL group was lower compared to the FRE (*p* = 0.001) and SRE (*p* = 0.012) groups (Table 2) (Figure 3).

### 3.3. Serum Total Protein and Albumin Concentration

**Figure 4:**
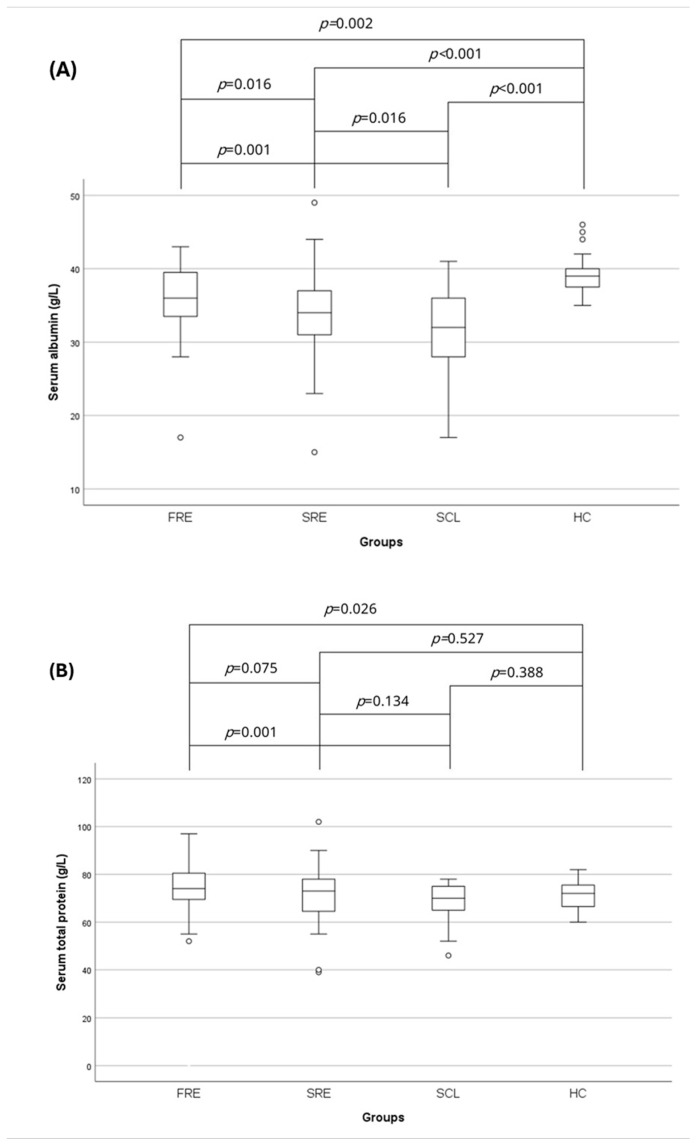
Box-plots comparing serum (**A**) albumin and (**B**) total protein concentrations in HC cats (*n* = 73) and cats with FRE (*n* = 59), SRE (*n* = 56) and intestinal SCL (*n* = 33), and (**B**) the serum total protein concentration in HC cats (*n* = 73) and cats with FRE (*n* = 58), SRE (*n* = 56) and intestinal SCL (*n* = 33). Circles represent outliers. Statistical significance was defined as *p* ≤ 0.05. FRE: food-responsive enteropathy; SRE: steroid-responsive enteropathy: SCL: small cell lymphoma; HC: healthy control cats.

The serum Alb concentration (g/L) of the HC group (median: 39.0; range: 29.0–48.0) was significantly higher compared to the FRE (median: 36.0; range: 17.0–43.0) (*p* = 0.002), SRE (median: 34.0; range: 15.0–49.0) (*p* < 0.001), and SCL (median: 32.0; range: 17.0–41.0) (*p* < 0.001) groups (Table 2) (Figure 4). The FRE group had significantly higher serum Alb concentration than the SRE (*p* = 0.016) and SCL (*p* = 0.001) groups (Table 2) (Figure 4). The serum TP concentration (g/L) was significantly lower in the HC group (median: 72.0; range: 60.0–82.0) compared to the FRE group (median: 74.5; range: 52.0–97.0) (*p* = 0.026) (Table 2) (Figure 4). Finally, the FRE group had a significantly higher serum TP concentration compared to the SCL group (median: 70.0; range: 46.0–78.0) (*p* = 0.001) (Table 2) (Figure 4).

### 3.4. Correlation of NLR, NMR, and LMR with Clinicopathologic Variables

In the FRE group, there was a statistically significant moderate negative correlation of NLR with serum Alb concentration (*ρ* (*n* = 59)  =  −0.482, *p* < 0.001). In addition, there was a statistically significant, weak, positive correlation of LMR with serum Alb concentration (*ρ* (*n* = 59)  =  0.329, *p* = 0.011). No other significant correlations were observed, including those between leukocyte ratios and the modified FCEAI score for each CE group.

### 3.5. NLR, NMR, and LMR and Disease Classification

**Figure 5:**
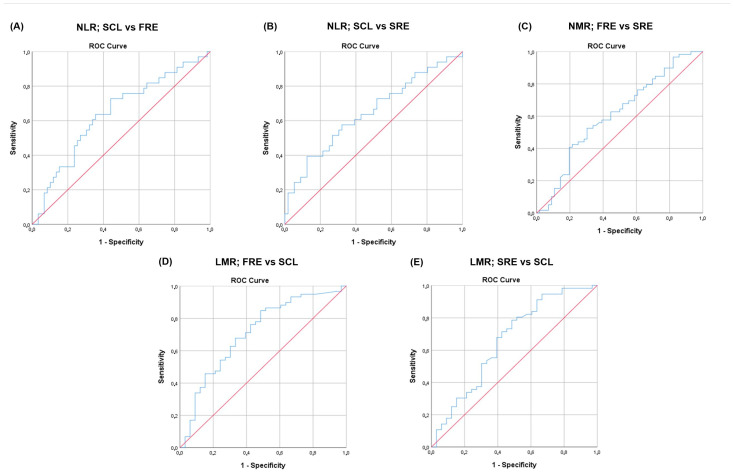
ROC curve of (**A**) NLR to differentiate cats with SCL from cats with FRE had an AUC of 0.64 (95% confidence interval [95% CI] = 0.522–0.759; *p* = 0.026). Using a cutoff value of 4.14, the sensitivity and specificity were 72.7% and 55.9%, respectively; (**B**) NLR to differentiate cats with SCL from cats with SRE had an AUC of 0.646 (95% CI = 0.524–0.767; *p* = 0.022). Using a cutoff value of 11.6, the sensitivity and specificity were 39.4% and 87.5%, respectively; (**C**) NMR to differentiate cats with FRE from cats with SRE had an AUC of 0.608 (95% confidence interval (95% CI = 0.505–0.712; *p* = 0.045). Using a cutoff value of 34.5, the sensitivity and specificity were 52.5% and 69.6%, respectively; (**D**) LMR to separate cats with FRE from cats with SCL had an AUC of 0.709 (95% CI = 0.596–0.823; *p* = 0.001). Using a cutoff value of 5.02, the sensitivity and specificity were 67.8% and 66.7%, respectively; (**E**) LMR to differentiate cats with SRE from cats with SCL had an AUC of 0.659 (95% confidence interval (95% CI = 0.536–0.782; *p* = 0.013). Using a cutoff value of 3.72, the sensitivity and specificity were 67.9% and 60.6%, respectively. The AUC was categorized as low (0.5 < AUC ≤ 0.7), moderate (0.7 < AUC ≤ 0.9), or high (0.9 < AUC ≤ 1.0). AUC: area under curve; NLR: neutrophil-to-lymphocyte ratio; NMR: neutrophil-to-monocyte ratio; LMR: lymphocyte-to-monocyte ratio; FRE: food-responsive enteropathy; SRE: steroid-responsive enteropathy; SCL: small cell lymphoma; ROC: receiver operating characteristics curve.

The area under the ROC (AUROC) for NLR that differentiated cats with SCL from cats with FRE was classified as low, at 0.64 (95% confidence interval [95% CI] = 0.522–0.759; *p* = 0.026) (Figure 5). Using a cutoff value of 4.14 for NLR, the sensitivity and specificity were 72.7% and 55.9%, respectively.

The AUROC for NLR to differentiate cats with SCL from cats with SRE was classified as low, at 0.65 (95% CI = 0.524–0.767; *p* = 0.022) (Figure 5). Using a cutoff value of 11.6 for the NLR, the sensitivity and specificity were 39.4% and 87.5%, respectively.

The AUROC for NMR to differentiate cats with FRE from cats with SRE was classified as low, at 0.61 (95% CI = 0.505–0.712; *p* = 0.045) (Figure 5). Using a cutoff value of 34.5 for the NMR, the sensitivity and specificity were 52.5% and 69.6%, respectively.

The AUROC for LMR to differentiate cats with FRE from cats with SCL was classified as moderate, at 0.71 (95% CI = 0.596–0.823; *p* = 0.001) (Figure 5). Using a cutoff value of 5.02 for the LMR, the sensitivity and specificity were 67.8% and 66.7%, respectively.

The AUROC for LMR to differentiate cats with SRE from cats with SCL was classified as low, at 0.66 (95% CI = 0.536–0.782; *p* = 0.013) (Figure 5). Using a cutoff value of 3.72 for the LMR, the sensitivity and specificity were 67.9% and 60.6%, respectively.

## 4. Discussion

Many studies have evaluated the use of leukocyte ratios as diagnostic and prognostic markers in humans with IBD [32,33,35,36,56,57,58,59]. In veterinary medicine, NLR has been shown to be useful as a diagnostic marker and to aid in the subclassification of CE in dogs [44]. To the authors knowledge, the present study is the first to compare selected leukocyte ratios among cats with FRE, SRE, and SCL, and to assess their diagnostic value as cost-effective and easily accessible biomarkers for subclassification of feline CE. Our results showed that NLR was significantly increased and NMR and LMR were decreased in all CE groups compared to the HC group. Moreover, NLR was significantly increased in cats with SCL compared to cats with FRE or SRE. However, the ROC analyses indicated that these biomarkers had suboptimal accuracy in discriminating between the different phenotypes of CE.

Although not definitively predictive of phenotype, several differences in NLR, NMR, and LMR were found between HC cats and cats having different phenotypes of CE. Different studies have investigated the utility of NLR as a diagnostic marker in dogs with CE and its correlation to clinical, laboratory, and histologic indices [44,46,49,60]. Summarizing the results from these canine studies, NLR was shown to aid the subclassification of dogs with CE based on the response to specific treatments and severity of clinical disease [44,45]. However, another study found that NLR had limited clinical utility as a biomarker for predicting treatment response [46]. In our study, NLR was significantly increased in all CE groups compared to the HC group, with the SCL group exhibiting significantly higher NLR values than the FRE and SRE groups. These findings underscore the central role of inflammation in all forms of CE. Inflammation is not only reflected in histopathology, which reveals infiltration by various inflammatory cells [48], but also in studies on cytokine expression in the intestinal mucosa [61,62,63]. Leukocytes have a key role in the inflammatory response seen in CE, with alterations in circulating leukocyte populations commonly observed, although limited data exist on their specific numbers in published research [64,65,66]. The increased neutrophil count across all CE groups compared to the HC group was the major component of the elevated NLR values. These increased neutrophil counts may be associated with the increased interleukin (IL)-8 expression (not assessed in the current study), which is the primary regulator of neutrophil responses and recruitment in the duodenal mucosa of cats with CE [61]. The decreased lymphocyte counts observed in the CE groups also contributed to the elevated NLR values in diseased cats relative to the HC group and could be attributed to chronic disease-related stress.

Two other leukocyte ratios, NMR and LMR, have been investigated in humans with IBD and systemic autoimmune diseases to evaluate their diagnostic utility and have been shown to be of value [67,68]. In veterinary medicine, studies evaluating NMR and LMR in different inflammatory or neoplastic diseases are lacking, with only one study focusing on canine CE [49,60,69,70]. In this canine study, monocyte-to-lymphocyte ratio (MLR) was useful in differentiating dogs with CE from healthy controls [49]. Several inflammatory and neoplastic diseases have been shown to affect leukocyte ratio values [41,42,43,71]. Other studies have investigated LMR or MLR in several different inflammatory conditions of cats, showing that these ratios are altered with infectious disease and systemic inflammation [43,71,72]. In the present study, both NMR and LMR were decreased between the CE and HC groups. This decrease in ratios is possibly related to the increased absolute monocyte counts found in CE cats compared to healthy cats.

The present study did not find a correlation between NLR, NMR, or LMR and clinical disease activity using the modified FCEAI score. Of interest, contradictory results regarding the correlation of leukocyte ratios to disease activity have been reported in CE and IBD affecting dogs and humans, as well as with canine pancreatitis [32,45,49]. In dogs with acute pancreatitis, both increased NLR and platelet-to-lymphocyte ratio were observed compared to healthy dogs. However, there was no association of these ratios with disease severity [73]. In the present study, the modified FCEAI score, based on commonly observed clinical signs, was highest in SCL cats, while cats with FRE had the lowest disease activity scores. Our results are in accordance with other studies, where the FCEAI has shown utility in defining clinical disease activity in cats with CE [2,65,74,75].

Differentiating FRE from SRE and SCL is of utility in clinical practice [9]. Having an easily accessible, time- and cost-effective marker to assist clinicians in prioritizing whether to initiate dietary trials or pursue more invasive diagnostic procedures (e.g., endoscopy and GI tract biopsy) would be beneficial. However, our findings indicate that the evaluated leukocyte ratios had low discriminatory power (low AUC values, all <0.75) and were not sufficient to distinguish CE phenotypes in cats. Therefore, these markers cannot substitute for more specific diagnostic methods such as histopathology (H&E), immunohistochemistry (IHC), or clonality testing (PARR).

This study has several limitations, with the primary one being its retrospective design. For example, the data obtained from the case files were not always available for such an analytical study (e.g., missing data on serum TP and Alb concentrations in HC cats). The interpretations from the medical records, including patient history, clinical examination findings, and the clinical scoring using the modified FCECAI score, were performed by different clinicians (AK and AJ). Although histopathologic examinations were performed by board-certified pathologists, review of all study intestinal biopsies by a single pathologist was not performed. Variability between pathologists in the interpretation of endoscopic biopsies using standardized grading criteria has previously been reported [76,77]. Additional limitations of this study include the fact that some cats in the SRE group only underwent a single diet trial, either prior to endoscopy or after receiving endoscopy and histopathology results. This was often necessitated by their poor condition, characterized by systemic clinical signs such as moderate to severe anorexia or decreased activity levels, particularly in older cats where a diagnosis of SRE or SCL was more likely. As a result, this may have led to some overlap among the CE subcategories, as multiple dietary trials are often required for accurate classification [78]. Furthermore, the majority of cats in the FRE group did not return to their previous diets. Consequently, some of these cases could represent food allergy or food intolerance rather than true FRE.

## 5. Conclusions

This retrospective study evaluated the diagnostic utility of NLR, NMR, and LMR in differentiating CE phenotypes in cats. While statistically significant differences in leukocyte ratios were observed between healthy cats and those with CE, as well as among CE subtypes, the overall diagnostic performance of these ratios was suboptimal. Despite being inexpensive and readily available, NLR, NMR, and LMR alone are insufficient to differentiate between CE phenotypes, particularly SRE and SCL, and cannot replace histopathology or advanced diagnostics such as IHC or molecular testing (e.g., PARR). Future prospective studies are warranted to investigate whether these leukocyte ratios may have prognostic value or predictive value regarding treatment response in cats with CE.

## Figures and Tables

**Table 1:** Signalment and disease activity of healthy control cats (HC) and cats with food-responsive enteropathy (FRE), steroid-responsive enteropathy (SRE), and intestinal small cell lymphoma (SCL) in the study.

Parameter		FRE (*n* = 59)		SRE (*n* = 56)		SCL (*n* = 33)		HC (*n* = 73)	
*Age (years)* *Median (range)* *Kruskal–Wallis test p* < 0.001		8 (1–16)		8.5 (1.5–17)		11 (6–16)		7 (1–15)	
FRE vs. HC (*Mann–Whitney test*) *p* = 0.170	SRE vs. HC (*Mann–Whitney test*) *p* = 0.008		SCL vs. HC (*Mann–Whitney test*) *p* < 0.001		FRE vs. SRE (*Mann–Whitney test*) *p* = 0.250		FRE vs. SCL (*Mann–Whitney test*) *p* < 0.001		SRE vs. SCL (*Mann–Whitney test*) *p* = 0.003
*Sex male/female (%)* *χ*^2^ test *p* = 0.053		31 (52.5)/28 (47.5)		31 (55.4)/25 (44.6)		18 (54.5)/15 (45.5)		25 (34.2)/48 (65.8)	
*Breed*									
DSH		33		42		28		61	
DLH		5		5		3		5	
Siamese		7		1				1	
Persian		3		2				1	
British Shorthair		2							
Russian Blue		1				1			
Ragdoll		1		1				1	
Norwegian Forest		1							
Scottish Fold		1							
Balinese		1							
Birman		1							
Snowshoe		1						2	
Devon Rex		1							
Siberian		1							
Leopard Bengal				1					
American Shorthair				1					
Exotic Shorthair				1					
Burmese				1					
Angora				1					
Maine Coon						1		2	
*FCEAI score; median (range)* *Kruskal–Wallis test p* < 0.001		2 (1–8)		4 (2–10)		5 (2–10)		0 (0–0)	
FRE vs. HC (*Mann–Whitney test*) *p* < 0.001	SRE vs. HC (*Mann–Whitney test*) *p* < 0.001		SCL vs. HC (*Mann–Whitney test*) *p* < 0.001		FRE vs. SRE (*Mann–Whitney test*) *p* < 0.001		FRE vs. SCL (*Mann–Whitney test*) *p* < 0.001		SRE vs. SCL (*Mann–Whitney test*) *p* = 0.002

FCEAI, feline chronic enteropathy clinical activity index (modified).

**Table 2:** Selected clinicopathologic data of healthy control cats (HC) and cats with food-responsive enteropathy (FRE), steroid-responsive enteropathy (SRE), and intestinal small cell lymphoma (SCL) included in the study.

Parameter	FRE (*n* = 59) Median (Range)	SRE (*n* = 56) Median (Range)	SCL (*n* = 33) Median (Range)	HC (*n* = 73) Median (Range)	K-W Test *p*-Value
WBC (×10^3^/μL)	10 (3.8–26.6)	8.54 (2.7–30.8)	11.02 (3.4–43.6)	6.8 (2.7–14.1)	<0.001
Neutrophils (×10^3^/μL)	6.8 (2.9–21.8)	5.7 (1.3–18.8)	8 (1.7–37.1)	4.3 (1.3–9.9)	<0.001
Lymphocytes (×10^3^/μL)	1.9 (0.1–9.0)	1.3 (0.1–8)	1.2 (0.1–10)	2.1 (0.1–6.3)	0.007
Monocytes (×10^3^/μL)	0.2 (0–0.8)	0.3 (0–1.9)	0.4 (0–8.2)	0.1 (0–1)	<0.001
NLR	3.6 (0.5–113)	4.8 (0.9–44.6)	8.3 (0.6–94.7)	2.2 (0.4–40)	<0.001
NMR	35 (8.3–482.5)	28.4 (4.3–718)	27.1 (0.5–94.7)	39.8 (5.7–276)	0.055
LMR	8.3 (0.5–60.1)	6 (0.7–88)	2.5 (0.3–100)	17 (1–341)	<0.001
Total protein (g/L)	75.0 (52.0–97.0) *	73.0 (39.0–102.0)	70.0 (46.0–78.0)	72.0 (60.0–82.0)	0.011
Albumin (g/L)	36.0 (17.0–43.0)	34.0 (15.0–49.0)	32.0 (17.0–41.0)	39.0 (29.0–48.0)	<0.001

K-W test: Kruskal–Wallis test; LMR: lymphocyte-to-monocyte ratio; NLR: neutrophil-to-lymphocyte ratio; NMR: neutrophil-to-monocyte ratio; WBC: white blood cells. * Serum total protein was measured in 58/59 FRE cats.

## Data Availability

The data are contained within this article.

## Supplementary Materials

The following supporting information can be downloaded at: https://www.mdpi.com/article/10.3390/vetsci12070613/s1, Table S1: Population characteristics and diagnosis of food-responsive enteropathy, steroid-responsive enteropathy and small cell lymphoma cases included in the study.

## Abbreviations

The following abbreviations are used in this manuscript:

Alb	albumin
AUC	area under the curve
AUROC	area under the ROC
CBC	complete blood count
CE	chronic enteropathies
FCEAI	feline chronic enteropathy activity index
FeLV	feline leukemia virus
FIV	feline immunodeficiency virus
fPLI	feline pancreatic lipase immunoreactivity
FRE	food-responsive enteropathy
GI	gastrointestinal
IBD	inflammatory bowel disease
IHC	immunohistochemistry
LMR	lymphocyte-to-monocyte ratio
NLR	neutrophil-to-lymphocyte ratio
NMR	neutrophil-to-monocyte ratio
PARR	PCR assay for antigen receptor rearrangement
PLR	platelet-to-lymphocyte ratio
ROC	receiver operating characteristic
SCL	small cell lymphoma
SRE	steroid-responsive enteropathy
TLI	trypsin-like immunoreactivity
TP	total protein
