# Supplementary material for: Blood Leukocyte Ratios as Predictive Markers of Chronic Enteropathy Phenotypes in Cats
Source: Vet Sci. 2025 Jun 24;12(7):613. doi: 10.3390/vetsci12070613 (PMC12298161; doi:10.3390/vetsci12070613)
Supplement: Supplementary file 1 [file vetsci-12-00613-s001.zip › vetsci-3677179-supplementary.pdf]

Supporting Information Table S1. Population characteristics and diagnosis of food-responsive enteropathy, steroid-responsive enteropathy and small cell lymphoma cases included in the study.

No.	Group/ Diagnosis	Breed	Age (years)	Sex	US	Histopathology	IHC	PARR
1	FRE	DLH	14	MN	NO	NO	NO	NO
2	FRE	DSH	16	FS	YES	NO	NO	NO
3	FRE	DSH	1.5	MN	NO	NO	NO	NO
4	FRE	DSH	3	MN	NO	NO	NO	NO
5	FRE	DSH	10	FS	NO	NO	NO	NO
6	FRE	DSH	13	FS	YES	NO	NO	NO
7	FRE	DLH	3	FS	YES	NO	NO	NO
8	FRE	DSH	1	MN	YES	NO	NO	NO
9	FRE	DSH	12	FS	YES	NO	NO	NO
10	FRE	DSH	5	MN	NO	NO	NO	NO
11	FRE	DSH	14	MN	YES	NO	NO	NO
12	FRE	DSH	10	FS	YES	NO	NO	NO
13	FRE	Devon Rex	5	FS	YES	NO	NO	NO
14	FRE	DSH	8	MN	YES	NO	NO	NO
15	FRE	DSH	11	FS	YES	NO	NO	NO
16	FRE	DSH	12	MN	YES	NO	NO	NO
17	FRE	DSH	8	MN	YES	NO	NO	NO
18	FRE	DSH	15	FS	YES	NO	NO	NO
19	FRE	DSH	15	FS	YES	NO	NO	NO
20	FRE	Siamese	9	MN	YES	NO	NO	NO
21	FRE	DSH	10	FS	YES	NO	NO	NO
22	FRE	DSH	3	MN	YES	NO	NO	NO
23	FRE	Snowshoe	10	FS	YES	NO	NO	NO
24	FRE	Birman	8	FS	YES	NO	NO	NO
25	FRE	DSH	9	FS	YES	NO	NO	NO
26	FRE	DLH	11	FS	YES	NO	NO	NO
27	FRE	DSH	11	FS	YES	NO	NO	NO
28	FRE	DLH	13	FS	NO	NO	NO	NO
29	FRE	DSH	9	FS	YES	NO	NO	NO
30	FRE	DSH	14	MN	YES	NO	NO	NO
31	FRE	DSH	15	MN	YES	NO	NO	NO
32	FRE	Balinese	7	MN	YES	NO	NO	NO
33	FRE	Ragdoll	14	MN	YES	NO	NO	NO
34	FRE	Persian	7	MN	YES	NO	NO	NO
35	FRE	Norwegian forest	1.5	M	YES	NO	NO	NO
36	FRE	Siamese	11	F	YES	NO	NO	NO
37	FRE	Scottish Fold	3	MN	YES	NO	NO	NO
38	FRE	DSH	5	MN	YES	NO	NO	NO
39	FRE	British Shorthair	2	FS	YES	NO	NO	NO
40	FRE	Siberian	2	MN	YES	NO	NO	NO
41	FRE	Siamese	11	MN	YES	YES	NO	NO

42	FRE	DSH	12	FS	YES	NO	NO	NO
43	FRE	DSH	11.5	MN	YES	YES	NO	NO
44	FRE	Siamese	7	FS	YES	NO	NO	NO
45	FRE	DSH	5	FS	YES	NO	NO	NO
46	FRE	DSH	5	FS	YES	YES	NO	NO
47	FRE	DSH	10	MN	YES	NO	NO	NO
48	FRE	DLH	3.5	MN	YES	NO	NO	NO
49	FRE	DSH	5	FS	NO	YES	NO	NO
50	FRE	DSH	3	MN	YES	NO	NO	NO
51	FRE	Siamese	3	F	NO	NO	NO	NO
52	FRE	Siamese	6	M	NO	YES	NO	NO
53	FRE	Siamese	1	F	NO	NO	NO	NO
54	FRE	Russian blue	6	MN	NO	NO	NO	NO
55	FRE	Persian	5	MN	YES	NO	NO	NO
56	FRE	Persian	7	MN	YES	YES	NO	NO
57	FRE	DSH	2	FS	YES	NO	NO	NO
58	FRE	British Shorthair	3.5	MN	YES	NO	NO	NO
59	FRE	DSH	3.5	MN	YES	NO	NO	NO
60	SRE	Persian	2	MN	YES	YES	NO	NO
61	SRE	Leopard Bengal	5	MN	YES	YES	NO	NO
62	SRE	DLH	14	FS	YES	YES	NO	NO
63	SRE	DSH	8	MN	YES	YES	NO	NO
64	SRE	DSH	10	MN	YES	YES	NO	NO
65	SRE	DLH	11	MN	YES	YES	NO	NO
66	SRE	DSH	6	FS	YES	YES	NO	NO
67	SRE	DSH	7	FS	YES	YES	NO	NO
68	SRE	DSH	8	MN	YES	YES	NO	NO
69	SRE	DSH	6	MN	YES	YES	NO	NO
70	SRE	DSH	15	MN	YES	YES	NO	NO
71	SRE	DSH	10	FS	YES	YES	NO	NO
72	SRE	DSH	11	MN	YES	YES	YES	NO
73	SRE	DSH	16	MN	YES	YES	NO	NO
74	SRE	DSH	13	MN	YES	YES	NO	NO
75	SRE	DSH	5	FS	YES	YES	NO	NO
76	SRE	DSH	6	MN	YES	YES	NO	NO
77	SRE	DSH	11	FS	YES	YES	NO	NO
78	SRE	DSH	3	MN	YES	YES	NO	NO
79	SRE	DSH	5	MN	YES	YES	NO	NO
80	SRE	DSH	16	MN	YES	YES	NO	NO
81	SRE	DSH	14	MN	YES	YES	NO	NO
82	SRE	DSH	5	FS	YES	YES	NO	NO
83	SRE	DSH	10	FS	YES	YES	YES	NO
84	SRE	DSH	15	MN	YES	YES	YES	NO
85	SRE	DSH	3	MN	YES	YES	NO	NO
86	SRE	DLH	10	FS	YES	YES	NO	NO
87	SRE	American Shorthair	7	MN	YES	YES	NO	NO

88	SRE	DLH	13	MN	YES	YES	NO	NO
89	SRE	Ragdoll	6	MN	YES	YES	NO	NO
90	SRE	DLH	14	FS	YES	YES	NO	NO
91	SRE	DSH	6	MN	YES	YES	NO	NO
92	SRE	DSH	10	MN	YES	YES	YES	NO
93	SRE	Exotic Shorthair	5	FS	YES	YES	NO	NO
94	SRE	Burmese	10	MN	YES	YES	NO	NO
95	SRE	DSH	9	FS	YES	YES	NO	NO
96	SRE	DSH	6	FS	YES	YES	NO	NO
97	SRE	DSH	10	FS	YES	YES	NO	NO
98	SRE	DSH	2	FS	YES	YES	NO	NO
99	SRE	Angora	16	FS	YES	YES	NO	NO
100	SRE	DSH	8	MN	YES	YES	NO	NO
101	SRE	DSH	7	FS	YES	YES	NO	NO
102	SRE	DSH	17	FS	YES	YES	NO	NO
103	SRE	DSH	7	FS	YES	YES	NO	NO
104	SRE	DSH	12	MN	YES	YES	NO	NO
105	SRE	DSH	11	MN	YES	YES	NO	YES
106	SRE	DSH	14	FS	YES	YES	NO	NO
107	SRE	Persian	1.5	MN	YES	YES	NO	NO
108	SRE	Siamese	11	FS	YES	YES	NO	NO
109	SRE	DSH	4	FS	YES	YES	NO	NO
110	SRE	DSH	1.5	FS	YES	YES	NO	YES
111	SRE	DSH	5	MN	YES	YES	NO	YES
112	SRE	DSH	8	MN	YES	YES	NO	NO
113	SRE	DSH	14	FS	YES	YES	NO	NO
114	SRE	DSH	3	FS	YES	YES	NO	NO
115	SRE	DSH	9	MN	YES	YES	NO	NO
116	SCL	DSH	8	FS	YES	YES	YES	NO
117	SCL	DSH	11	MN	YES	YES	YES	NO
118	SCL	DSH	10	FS	YES	YES	YES	NO
119	SCL	DSH	10	MN	YES	YES	YES	NO
120	SCL	DSH	14	MN	YES	YES	YES	NO
121	SCL	DSH	11	MN	YES	YES	YES	NO
122	SCL	DSH	14	MN	YES	YES	YES	NO
123	SCL	DSH	10	FS	YES	YES	YES	NO
124	SCL	DSH	16	FS	YES	YES	YES	NO
125	SCL	DSH	14	MN	YES	YES	YES	NO
126	SCL	DSH	11	FS	YES	YES	YES	NO
127	SCL	DSH	14	MN	YES	YES	YES	NO
128	SCL	DSH	14	MN	YES	YES	YES	NO
129	SCL	DSH	11	MN	YES	YES	YES	NO
130	SCL	DSH	12	FS	YES	YES	YES	NO
131	SCL	DSH	11	FS	YES	YES	YES	NO
132	SCL	DSH	15	MN	YES	YES	NO	NO
133	SCL	Maine Coon	9	FS	YES	YES	YES	NO
134	SCL	DSH	12	MN	YES	YES	NO	NO
135	SCL	DSH	11	FS	YES	YES	YES	NO

136	SCL	DSH	10	MN	YES	YES	YES	NO
137	SCL	DSH	6	FS	YES	YES	YES	NO
138	SCL	DSH	13	FS	YES	YES	YES	NO
139	SCL	DLH	14	MN	YES	YES	YES	NO
140	SCL	DLH	7	MN	YES	YES	YES	NO
141	SCL	DLH	8	MN	YES	YES	YES	NO
142	SCL	DSH	8	MN	YES	YES	YES	NO
143	SCL	Russian bluE	12	MN	YES	YES	NO	NO
144	SCL	DSH	8	FS	YES	YES	NO	YES
145	SCL	DSH	12	FS	YES	YES	NO	NO
146	SCL	DSH	14	FS	YES	YES	NO	YES
147	SCL	DSH	13	FS	YES	YES	NO	NO
148	SCL	DSH	9	MN	YES	YES	NO	YES

Abbreviations: F: Female, FS: Female spayed, FRE: Food-responsive enteropathy, IHC: Immunohistochemistry, M: Male, MN: Male neutered, NO: Did not perform, PARR: PCR assay for antigen receptor rearrangement, SCL: Small cell lymphoma, SRE: Steroid-responsive enteropathy, US: Ultrasonography, YES: Performed
